# Sand fly–associated phlebovirus with evidence of neutralizing antibodies in humans and dogs in Kosovo

**DOI:** 10.1080/22221751.2025.2608407

**Published:** 2025-12-22

**Authors:** Elif Kurum, Xhevat Jakupi, Betim Xhekaj, Katharina Platzgummer, Ina Hoxha, Julia Walochnik, Vít Dvorák, Donjeta Hajdari, Pranvera Abazi, Adelheid G. Obwaller, Jovana Stefanovska, Aleksandar Cvetkovikj, Kurtesh Sherifi, Remi Charrel, Edwin Kniha, Nazli Ayhan

**Affiliations:** a Unité Des Virus Émergents (UVE: Aix-Marseille Univ, Università di Corsica, IRD 190, Inserm 1207, IRBA), Marseille, France; b Medical Faculty, University of Prishtina “Hasan Prishtina”, Prishtina, Kosovo; c Faculty of Agriculture and Veterinary, University of Prishtina, Prishtina, Kosovo; d Center for Pathophysiology, Infectiology and Immunology, Institute of Specific Prophylaxis and Tropical Medicine, Medical University Vienna, Vienna, Austria; e Department of Parasitology, Faculty of Science, Charles University Prague, Prague, Czech Republic; f National Institute of Public Health of Kosovo, Prishtina, Kosovo; g Division of Science, Research and Development, Federal Ministry of Defence, Vienna, Austria; h Department of Parasitology and Parasitic Diseases, Faculty of Veterinary Medicine-Skopje, Ss. Cyril and Methodius University in Skopje, Skopje, North Macedonia; i National Reference Center for Arboviruses, Inserm-IRBA, Marseille, France

**Keywords:** Phlebovirus, sandfly-borne phleboviruses, Republic of Kosovo, arbovirus, bunyavirales

## Abstract

The Balkan Peninsula is a hotspot for sand fly-borne phleboviruses (SbPVs), yet Kosovo had no confirmed viral detection invectors despite serological evidence of human and animal exposure. This study reports the discovery, genetic characterization, and seroprevalence of a novel phlebovirus, Grapi virus (GRPV), in Kosovo. Entomological surveys (2022–2023) collected 3,575 sand flies across seven districts. Morphological and molecular identification revealed *Phlebotomus perfiliewi* as the dominant species. Pan-phlebovirus RT–PCR screening identified GRPV in seven pools. Complete genome sequencing confirmed its tripartite genome, sharing 97.55-98.70% nucleotide identity with Bregalaka virus, classifying it within the Phlebovirus adanaense species. Phylogenetic analysis revealed segment-specific ancestry, suggesting recombination events between Bregalaka virus, Adana virus, and Medjerda Valley virus. Seroprevalence studies using neutralization assays detected GRPV-specific antibodies in 13.0% of humans and 2.7% of dogs. Human seropositivity peaked in adolescents and declined with age, while dogs showed higher rates in purebred and unhealthy ones. No cross-reactivity with Toscana or Sicilian viruses was observed, indicating distinct immunological responses. GRPV replicated efficiently in Vero cells and more slowly in mosquito cells, suggesting mammalian adaptation. GRPV detection in Kosovo underscores the role of the Balkan region in SbPV emergence. GRPV zoonotic potential is supported by the anthropophilic feeding behaviour of *Ph. perfiliewi* and by the significant seroprevalence rates in dogs and humans. Limitations include biased human/dog sampling and sparse northern Kosovo coverage. Investigating GRPV pathogenicity and ecology; integrated surveillance and diagnostics are essential for the future.

## Introduction

A growing number of studies indicate that the Balkan Peninsula is a critical region for the emergence and transmission of vector-borne diseases (VBDs) due to its geographical location as a cross-border region between Asia and Europe [1]. The “sandfly fever” disease, caused by two sandfly-borne phleboviruses (SbPVs), the sandfly fever Sicilian virus [SFSV; *Phlebovirus siciliaense*] and the sandfly fever Naples virus [SFNV; *Phlebovirus napoliense*], was first documented in the Balkan peninsula at the end of the nineteenth century [2].

Recent sand fly surveillance studies have identified known and new phleboviruses circulating in the Balkans [1]. Balkan virus (BALKV) was detected in Croatia, Albania and Bosnia and Herzegovina [3] in *Phlebotomus neglectus* sand flies. Corfou virus (CFUV; *Phlebovirus corfouense*) was isolated in Greece from *Ph. neglectus* [4] and Adria virus (ADRV) was detected in sand flies in Albania and in a child with fever in Greece [5]. Recently, Zaba virus (ZABAV) was isolated in *Ph. neglectus* from Croatia and Bregalaka virus (BREV) was isolated in *Ph. perfiliewi* from North Macedonia [6]. In addition, Toscana virus (TOSV; *Phlebovirus toscanaense*), which causes febrile illnesses and neurological diseases, has been detected in *Ph. neglectus* from Croatia [7]. The circulation of pathogenic SbPVs such as SFSV and TOSV was also demonstrated by sero-epidemiological studies in humans in Greece and Croatia [8,9]. Together, these results demonstrate that the Balkan area is a hotspot for SbPVs.

Although no SbPVs have been identified in sand flies of Kosovo, seroprevalence studies have shown high rates of SbPV antibody rates in Austrian soldiers serving in the country [10] and TOSV and SFSV neutralizing antibodies in domestic animals such as sheep, cattle and dogs [11,12].

Kosovo’s humid continental climate creates favourable conditions for the proliferation of sand flies. To date, nine species have been identified in the country [13]. A recent entomological study showed predominant presence and high abundance of *Ph. neglectus* and *Ph. perfiliewi* in the climatically favourable regions of Kosovo [14].

Here we present the identification, isolation and genetic characterization of a new virus (Grapi virus (GRPV), genus *Phlebovirus*) from sand flies collected in Kosovo. The complete genome of GRPV was sequenced and virus replication was tested in mammalian and mosquito cells. Human and canine serum samples from Kosovo provided evidence of infection based on neutralization assays using GRPV.

## Material and methods

### Sand fly trapping and morphological identification

In 2022 and 2023, two independent entomological surveys using CDC miniature light traps (John W. Hock Company, Gainesville, FL, USA) were conducted in all seven districts of the Republic of Kosovo, resulting in 160 sampling sites and 3,575 captured sand flies as published elsewhere [14,15]. Traps were set at 1–2 h before sunset (6–8 pm) and collected around sunrise (7–9 am). After collection, the nets were placed immediately in dry ice. For a time-efficient process to avoid RNA/DNA degradation, morphological identification was performed individually [16] at locations with less than 100 trapped specimens, resulting in 1,452 individually identified specimens. For the remaining 2,123 specimens originating from only five locations, a fraction (minimum of 8%) of the catches per location was identified (total of 372 specimens) and the rest (1751 specimens) were pooled [up to 30 specimens] by location, sex, and feeding status. For morphological identification, specimens were kept at −80°C until head and terminal segments of the abdomens were dissected and subsequently slide-mounted in CMCP-10 mountant (Polysciences, Inc., Warrington, PA, USA). Identification was based on published morphological keys and descriptions of male genitalia, female spermatheca, and pharyngeal armature [14,17]. The remaining body parts were immediately transferred to separate tubes for homogenization and kept at −80°C until further processing.

### Molecular identification of sand fly species

Molecular identification of sand fly species in virus positive pools was conducted using PCR-based barcoding targeting the *cytochrome c oxidase subunit I* (COI) gene as previously explained [6]. The resulting PCR products were purified following standard protocols and sequenced using next-generation sequencing (NGS). The obtained sequences were analyzed by comparing them with reference sequences in the GenBank database.

### Processing pools of sand flies and virus detection

Tubes containing individuals or pools of sandflies were suspended in 500 µL (individual sand flies and pools with up to 15 specimens) or 1000 µL (pools with more than 15 specimens) of Dulbecco’s Modified Eagle Medium (DMEM) supplemented with 20% bovine serum albumin, 1% penicillin/streptomycin, 10 µg/mL gentamicin, and 0.25 µg/mL amphotericin B (all from Gibco, Thermo Fisher Scientific). Two metal beads (3 mm diameter) were added to each 2.0 mL tube and homogenized with a TissueLyser bead mill (QIAGEN GmbH, Hilden, Germany) for 1 min of shaking at 30 Hz. The homogenate was cleared via centrifugation in a 4 °C benchtop centrifuge for 5 min at 14,000 rpm.

For RNA isolation, up to twenty homogenates (20 µL each) of individually identified and homogenized sand flies were pooled by sex, feeding status, species and location, and are herein referred to as super-pools. While 200 µL of the supernatant of pooled males, females, and engorged females were used for RNA isolation, all with a QIAmp® RNeasy Mini kit 250 (Qiagen, Hilden, Germany) strictly following the manufacturer’s protocol with a final elution volume of 50 µL.

All RT–PCR reactions were performed using 5 µL of nucleic acid eluate. The samples were tested by using a pan-phlebovirus RT–PCR assay [18]. Thermal-cycling program consisted of 50 °C for 30 min and 94 °C for 2 min, followed by 40 cycles at 94 °C for 30s, the annealing temperature 55 °C for 1.5 min, and 68 °C for 30 s, with a final elongation steps at 68 °C for 7 min then hold 20 °C for 2 min. Following electrophoresis on a 2% agarose gel, the PCR products were visualized under UV illumination. Positive PCR products were sequenced via Next-Generation Sequencing (NGS).

After virus identification, RNA of stored homogenates of pools positive for Phlebovirus RNA were re-analyzed individually by mixing 200 µL of sand fly homogenate with 200 µL of VXL lysis buffer (Qiagen, Hilden Germany) before nucleic acid extraction on the BioRobot EZ1-XL Advanced with the Virus Extraction Mini Kit [Qiagen]. Thereafter, all pan-phlebovirus positive samples were re-tested using a BREV specific real-time RT-qPCR [6]. Following the real-time RT–PCR analysis, the minimum infection rate (MIR) was calculated under the assumption that only a single individual was positive within any given positive pool using the formula (number of positive pools/total specimens tested) × 1,000 [19].

### Virus isolation

A volume of 50 µL of homogenized sand fly material was suspended in 350 µL of enriched Minimum Essential Medium [MEM] containing 1% L-glutamine, non-essential amino acids, Penicillin–Streptomycin [200Mm], and 3% Amphotericin B and inoculated into Vero E6 cells. After incubation at 37°C in a 5% CO_2_ atmosphere for 1 h, 2.5 ml of enriched MEM containing 5% fetal bovine serum [FBS] with aforementioned antibiotics were added. The cell cultures were monitored daily for the presence of cytopathic effects and passaged three times consecutively. Following each passage, 200 μl of supernatant were tested using the standard RT–PCR protocols. Virus stock solution was produced after passage 1 and used for all further experiments. The infectious titre was determined using a 50% tissue culture infectious dose (TCID₅₀) assay. Ten-fold serial dilutions of the viral stock, ranging from 10^−^¹ to 10^−^¹², were prepared and inoculated onto confluent Vero E6 cells seeded in a 96-well plate. Viral titres were subsequently calculated following the method of estimating fifty-percent endpoints [20].

### Complete genome sequencing

The GRPV SP59 viral stock passage 1 was selected for complete genome sequencing using NGS. A total of 200 µL of cell culture supernatant medium was incubated at 37 °C for 1 h with 30 U of Benzonase (Novagen) and MgCl_2_. RNA extraction was performed using the QIAampViral RNA Mini Kit (Qiagen, Hilden, Germany) on the BioRobot EZ1-XL Advanced (Qiagen, Hilden Germany). Random tagged primers were used for random amplifications with RT–PCR (Applied Biosystems, Waltham, MA, USA). The PCR products were purified (Amicon ultracentrifugal filters; Millipore), and 200 ng was used for sequencing using the Ion PGM sequencer (Life Technologies SAS, Saint Aubin, France). CLC Genomics Workbench 7.0.4 was used to process reads. Reads longer than 30 nts were trimmed using the CLC Genomics Workbench with 99% quality per base and mapped to reference sequences (BREV, GenBank acc no: MG573144, MG573145, MG573146 respectively for the L, M and S segments). Reads that mapped to at least 50% of the reference sequence with a minimum identity of 80% were used. Specific primers were designed to complete sequence gaps and purified PCR products were sequenced by NGS.

### Genetic and phylogenetic analysis

To determine the phylogenetic relationship of the novel sand fly-associated phlebovirus, GRPV, with other members of the *Phlebovirus* genus, the L, M, and S gene sequences were aligned using ClustalW, along with homologous sequences from selected phleboviruses retrieved from GenBank. Phylogenetic trees were inferred based on the complete amino acid sequences of the RNA-dependent RNA polymerase, glycoproteins N and C, nucleoprotein and non-structural proteins, employing maximum likelihood analysis with the LG substitution model in MEGA6 software. Statistical support of the tree topology was evaluated by bootstrap resampling of the sequences 1,000 times.

The Open Reading Frame sequences of the three segments for GRPV and other closely related viruses were combined and examined for potential recombination events using the RDP4 software [21], which includes the RDP, GENECONV, Bootscan, MaxChi, Chimaera, SiScan, and 3Seq methods. Following the detection of a recombination signal using these methods, RDP4 estimates the approximate breakpoint positions with a hidden Markov model (BURT), and subsequently identifies the recombinant sequence using the PHYLPRO, VISRD, and EEEP methods [21]. All methods were run using their default program settings. Additional genomic similarity analysis was performed in SimPlot++ using the Jukes-Cantor model. GRPV was used as the query sequence and was compared with genetically closest viruses. Coding regions (L, Gn, Gc, N, NSs) were annotated to inspect region-specific similarity patterns.

### Dog and human serum samples

Dog serum samples were collected from dogs between summer 2021 and spring 2022 in the frame of a *Leishmania* seroprevalence study in Kosovo as previously explained [22], also recording age, sex, breed, and health status information.

Human serum samples were collected from two different laboratories during period of time September – November 2024: Lab 1, a specialized HIV/Hepatitis laboratory, and Lab 2, which primarily handled samples requested for TORCH testing (Toxoplasma, Rubella, Cytomegalovirus, HSV2), along with some respiratory pathogen tests. Samples were randomly selected from both laboratories, ensuring a balanced distribution in terms of geographic origin.

### Seroneutralization [dogs and human]

To test whether GRPV infects humans and dogs, we analyzed 288 dog and 598 human serum samples using seroneutralization assay.

Serum samples were heat-inactivated at 56°C for 30 min and diluted 4 times (2-fold dilution) in a 96-well microplate. The contact step, during which specific antibodies bind to infectious virus particles and can inhibit virus growth, was performed for one hour at 37°C in a 100μL volume using 100TCID_50_ GRPV. A 100μL volume of Vero E6 cell suspension (5 × 10⁵ cells/mL) was added to the serum-virus mixture. Negative and positive controls were included in each microplate. All dilution and dispensing steps as well as cytopathic effect (CPE) analysis (evaluation of the presence or absence of a CPE) were automated by using epMotion 5075 (Eppendorf, Hamburg, Germany) and Incucyte SX5 Live-Cell Analysis (Sartorius, Göttingen, Germany) in a NSB3 laboratory. On day 5 post-infection, the plates were examined, and the positivity was determined using a threshold value of ≥40 titre for calculation of the overall seroprevalence rate.

Dog sera were previously tested for TOSV and SFSV using the same seroneutralization technique [12], and thus, cross reactivity between the tested viruses could be determined.

### Statistical analysis and mapping of prevalence

Data were prepared with Microsoft Excel for Mac and analyzed with RStudio for Mac (R Core Team 2023). Categorical data [age, breed, district, health status, GRPV seroprevalence, and sex] was analyzed by Fisher’s exact test with overall prevalence as the predictor variable. Odds ratios (OR) with exact 95% confidence intervals (CI) were estimated. On a municipality level, we refrained from statistical analysis due to the partially low number of available samples. A two-sided *p*-value <0.05 was considered statistically significant. Prevalence was mapped with QGIS (QGIS Development Team, 2019) using first-level administrative divisions of Kosovo (year 2015) taken from https://earthworks.stanford.edu/catalog/stanford-zh532mm5047.

All seroneutralization assay data were analyzed using GraphPad Prism (v9.4.1) on Windows. Spearman’s rank correlation test was used to assess associations between neutralization titres of TOSV, SFSV, and GRPV using a two-tailed approach. Significance was set at *p* < 0.05, with results reported as Spearman’s ρ, *p*-values, and 95% confidence intervals.

### *In vitro* viral growth kinetics

Confluent monolayers of mammalian (Vero African green monkey kidney cells, Vero-ATCC CCL81) and mosquito (*Aedes albopictus* larvae cells, C6/36, ATCC CRL-1660) cell lines were infected with GRPV at multiplicities of infection [MOI] of 0.01, 0.1, and 1 in duplicate. Aliquots of infectious cell culture supernatants were collected every 24 h for an 11-day period. Clarified virus supernatants were then extracted using the QIAcube (Qiagen). Viral genome copies were quantified by real-time RT–PCR, using plasmid-based quantification standards. The viral genome copy number was quantified using real-time RT–PCR with plasmid-based quantification standards.

## Results

### Sand fly trapping and identification

Of 3,575 sand flies trapped in the two surveys, 1,452 individually identified specimens showed eight species of two genera (Table 1). Of the remaining 2,123 specimens originating from only five locations, 372 specimens were morphologically identified, revealing 330 (88.7%) *Ph. perfiliewi* and 42 (11.3%) *Ph. neglectus* specimens, and no other species [14,15].

**Table 1.** Combined number of individually identified sand fly species of both surveys [14,15].

Species	Female (engorged)	Male	Total
*Ph. perfiliewi*	651 (110)	65	716
*Ph. neglectus*	394 (126)	269	663
*Ph. tobbi*	23 (2)	8	31
*Ph. simici*	19 (4)	3	22
*Ph. balcanicus*	5 (0)	4	9
*Ph. papatasi*	1 (0)	1	2
*Ph. mascittii*	1 (0)	0	1
*S. minuta*	7 (2)	1	8
Total	1101 (244)	351	1452

### Virus detection and isolation

All 3,575 sand flies were analyzed in 80 pools (containing up to 30 specimens) and 120 super-pools (containing up to 20 specimens)[14,15]

**Figure 1.**
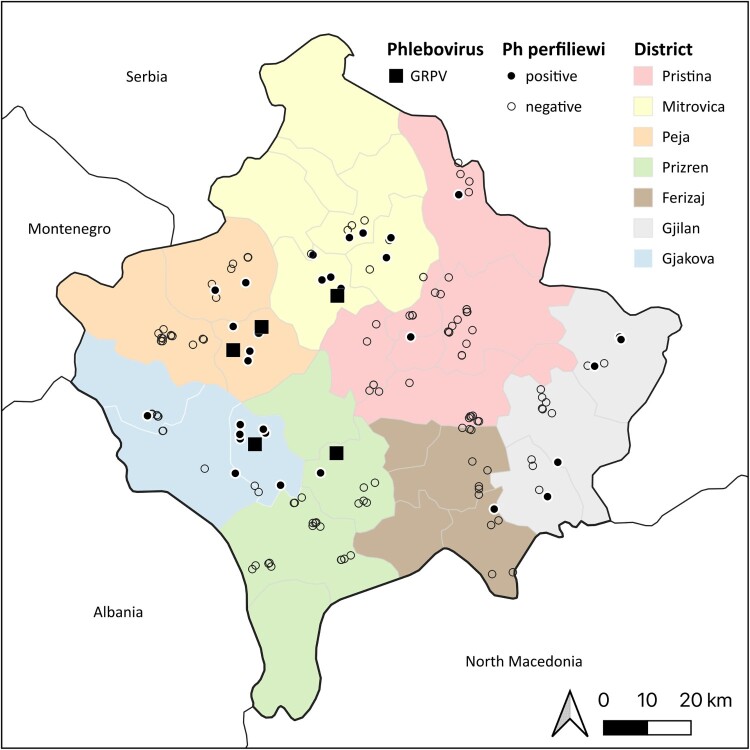
Mapped trapping sites with GRPV detection and *Ph. perfiliewi* occurrence. First-level administrative divisions of Republic of Kosovo were obtained from https://earthworks.stanford.edu/catalog/stanford-zh532mm5 047 (accessed on 18 July 2023).

Of 80 pools and 120 super-pools tested, six pools (SP1, SP17, SP30, SP44, SP59, SP65) with 30 specimens and one super-pool (P3/110) with 10 engorged specimens, respectively, were positive using the pan-Phlebovirus assay and the specific real time RT-qPCR assay with Ct values shown in Table 2, accounting for a MIR of 1.96. The positive samples originated from four districts, namely Mitrovica, Peja, Prizren, and Gjakova (Figure 1).

**Table 2.** Grapi virus RT-qPCR-positive samples.

Sample	Sample size	District (code)	Sand fly species	Collection year	Isolation	Ct values
P3/110^a^	10 engorged females	Peja (03)	*Ph. perfiliewi*	2022	yes	32.87
SP1	30 females	Mitrovica (02)	*Ph. perfiliewi*	2022	no	33.78
SP17	30 females	Gjakova (07)	*Ph. perfiliewi*	2022	no	30.63
SP30	30 females	Peja (03)	*Ph. perfiliewi*	2022	no	32.84
SP44	30 females	Peja (03)	*Ph. perfiliewi*	2022	no	31.96
SP59	30 females	Prizren (04)	*Ph. perfiliewi*	2022	yes	26.83
SP65	30 females	Prizren (04)	*Ph. perfiliewi*	2022	yes	25.44

^a^ Super-pool, individually re-tested with one specimen positive.

The two samples (SP59 and SP65) displaying the lowest Ct value [e.g. the highest viral loads] (Supplementary Table 2) and all 10 individual samples of super-pool P3/110 were then inoculated on Vero E6 cell monolayers. SP59, SP65, and one sample of P3/110 showed a clear cytopathic effect at day 3 after inoculation (Supplementary Figure 1). The virus was named Grapi virus (GRPV) after the Grapi forest in the Prizren region, where the SP59 strain originated. Virus replication in Vero E6 cells was confirmed by testing cell culture supernatant with the GRPV RT-qPCR assay, which showed active viral replication passage after passage. The SP59 strain was used for seroneutralization assays and for viral growth kinetic studies. In addition, the SP59 strain was produced and qualified based on requirements of the European Virus Archive catalog where it is accessible for the scientific community (https://www.european-virus-archive.com) with the following codes: UVE/GRPV/2022/XK/SP59 (Ref-SKU: 001V-06224).

### Sand fly species molecular identification

The blast results of the COI sequences corresponding to the seven GRPV positive samples revealed >99% identity with *Ph. perfiliewi* reference sequences in GenBank. Moreover, our sequences were most closely related to those corresponding to previously obtained sequences from Kosovo [GenBank acc no PP296457] and Greece [GenBank acc no KU519504].

### Complete genome sequencing

The complete genome sequence of strain SP59 was obtained from cell culture supernatant. GRPV displays the tripartite genome structure characteristic of phleboviruses. Its large segment (L) encodes the RNA-dependent RNA polymerase (RdRp, 6288-nt open reading frame [ORF] [2096 aa]) (GenBank acc no PV368852). The medium segment (M) encodes a glycoprotein precursor (GPC, 4008-nt ORF [1336 aa]), which is post-translationally cleaved into two viral surface glycoproteins (Gn and Gc) and a non-structural M protein (NSm) (GenBank acc no PV368853). The small segment (S) encodes a nucleocapsid protein (N) of 746 nt (248 aa) and a non-structural S protein (NSs) of 858 nt (286 aa) (GenBank acc no PV368854).

The highest pairwise nucleic acid identity between GRPV and BREV was 98.70% for the L segment, 97.55% for the M segment and 98.46% for the S segment. According to the ICTV criteria for species delineation [23], GRPV is a new virus that should be included in the *Phlebovirus adanaense* species together with BREV, Adana virus [ADAV], and Ponticelli virus [PONV].

Protein sequence similarity analysis showed that the GRPV displays a very high level of similarity to BREV. The L segment–encoded RdRp; shares 99.62% identity, the M segment–encoded GPC shares 98.95% identity, and the S segment–encoded N and NSs proteins share 99.19% and 100% identity, respectively, with those of BREV.

The pairwise distance data have been presented in Supplementary Table 1.

### Genetic and phylogenetic analysis

**Figure 2.**
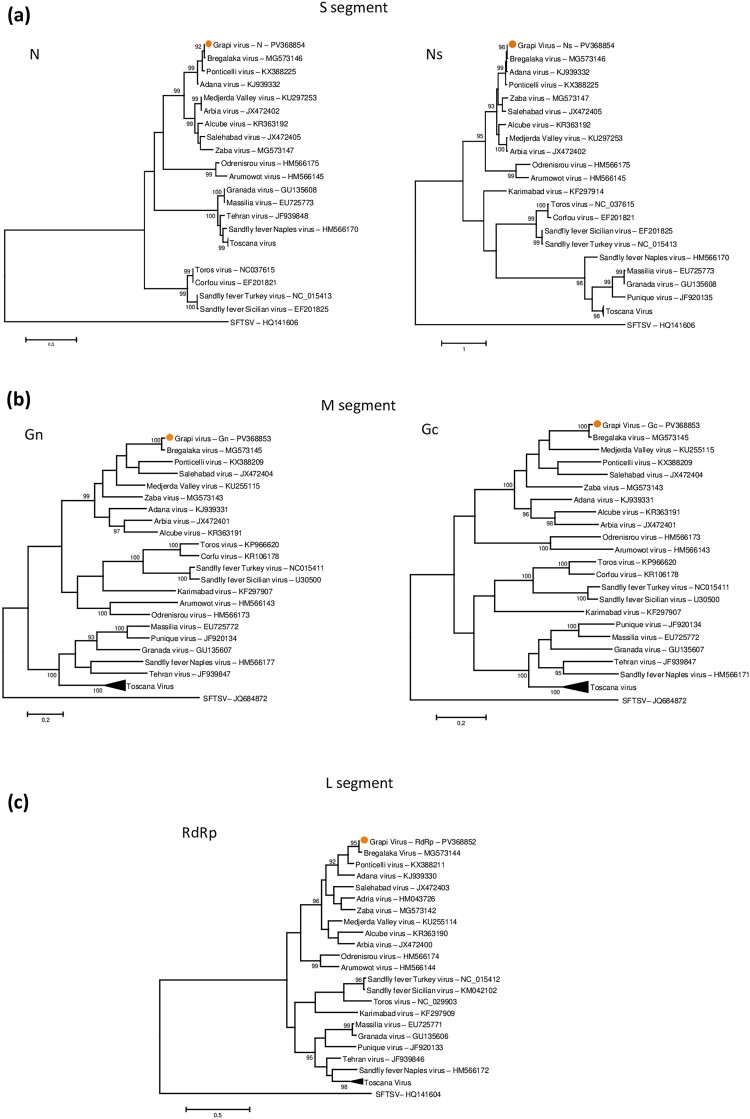
The phylogenetic relationships of phlebovirus amino acid sequences were analyzed based on the nucleocapsid and non-structural protein N (a), the glycoprotein N and C (b), and the RNA-dependent RNA polymerase (RdRp) (c).

Phylogenetic analysis showed that the L and S segments of GRPV have similar topologies and are closely related to BREV (GenBank accession no. MG573144 and MG573146), ADAV (GenBank acc. no. KJ939332 and KJ939330) and PONV (GenBank accession no KY354387 and KX388223) (Figure 2).

Based on the analysis of the partial genetic distances of the amino acids of the L segment, SP59 and SP65 were more closely related to each other than to 3/110 (0.1-0.7%).

Recombination detection analysis performed using RDP4 suggested that, for the L and S segments of GRPV, ADAV and Medjerda Valley virus (MVV) represented the major and minor parental viruses, respectively (Supplementary Figure 2). However, the M segment appeared to present the opposite pattern with possible recombination breakpoints. These results may explain the topological differences observed in Figure 2. Results from SimPlot++ were congruent with the RDP4 recombination analysis, with regions showing sharp similarity drops in the M segment overlapping the putative recombination breakpoints identified by RDP4. This concordance supports the recombinant nature of the M segment (Supplementary Figure 3).

### Seroneutralization of dog and human sera

Of 288 dog sera, 8 (2.7%; 95% CI: 1.3–5.6) showed neutralizing antibodies (NT-Ab) against GRPV, with NT titres ranging from 1:40 (5 sera) to 1:80 (3 sera). If the titres of 1:20 (21 sera) are included, the overall positivity rate is 10.1% (29/288, 95% CI: 7.0–14.3) (Supplementary Table 3). We can conclude that GRPV can infect dogs and that the prevalence rate is ranging from 2.7 to 10.1% in dogs depending upon the stringency of the retained cut-off value.

No significant difference was observed between the seroprevalence rates and most of the factors analyzed, but the prevalence rates doubled in healthy dogs (2.4%, 95% CI: 1.0–5.5) compared to unhealthy dogs (4.8%, 95% CI: 0.8–17.4), and almost tripled in mixed-breed dogs (1.7%, 95% CI: 0.4–5.3) compared to purebred dogs (4.5%, 95% CI: 1.7–10.7). Seroprevalence increased with age, being lowest in dogs aged 0 to 4 (1.8%, 95% CI: 0.5–5.6), followed by those aged 4 to 8 years (3.3%, 95% CI: 0.9–10.0) and those aged over 8 years (6.3%, 95% CI: 1.1–22.2) (Table 3). No association was found between dogs with leishmaniasis and those without.

**Table 3.** Association of GPRV seroprevalence in dogs with various risk factors.

Parameter (sample size)	Positive (%)	OR (95% CI), *P-*value
Sex		
Female (147)	4 (2.7%)	Reference
Male (141)	4 (2.8%)	1.04 (0.2–5.7), 1
Health status		
Normal (246)	6 (2.4%)	Reference
Disrupted (42)	2 (4.8%)	2.0 (0.2–11.7), 0.3
Breed		
Mixed (177)	3 (1.7%)	Reference
Pure-bred (111)	5 (4.5%)	2.7 (0.5–18.0), 0.3
*Leishmania*		
Negative (275)	8 (2.9%)	Reference
Positive (13)	–	–
Age group		
0–4 years (165)	3 (1.8%)	Reference
4–8 years (91)	3 (3.3%)	1.8 (0.2–14), 0.7
>8 years (32)	2 (6.3%)	3.6 (0.3–32.5), 0.2

Of 598 human sera, 78 (13.0%, 95% CI: 10.5–16.1) showed neutralizing antibodies (NT-Ab) against GRPV, with NT titres ranging from 1:40 (69 sera, 11.5%, 95% CI: 9.1–14.4) to 1:80 (9 sera, 1.5%, 95% CI: 0.7–2.9) (Supplementary Table 3). No significant difference was observed between women and men. Seroprevalence varied according to age group, being highest among 11 to 20 year-olds (27.3%, 95% CI: 13.9–45.8) and lowest among 51 to 60 year-olds (2.8%, 95% CI: 0.5–10.7). In general, seroprevalence was higher among the under 40 years of age than among those over 40 years of age (Table 4).

**Table 4.** GPRV seroprevalence in humans associated with sex and age group.

Parameter (sample size)	Positive (%)	OR (95% CI), *P-*value
Sex		
Female (414)	55 (13.3%)	Reference
Male (184)	23 (12.5%)	0.9 (0.5–1.6), 0.9
Age group		
1–10 years (33)	7 (21.2%)	Reference
11–20 years (33)	9 (27.3%)	1.3 (0.4–5.1), 0.8
21–30 years (159)	23 (14.5%)	0.6 (0.2–1.9), 0.4
31–40 years (143)	21 (14.7%)	0.6 (0.2–2.0), 0.4
41–50 years (67)	8 (11.9%)	0.5 (0.1–1.8), 0.2
51–60 years (71)	2 (2.8%)	0.1 (0.01–0.6), 0.004
61–70 years (63)	5 (7.9%)	0.3 (0.07–1.3), 0.1
>70 years (29)	3 (10.3%)	0.4 (0.07–2.2), 0.3

All the raw seroneutralisation data for dog and human have been presented in Supplementary Tables 6 and 7.

The analysis of Spearman's rank correlations evaluated the relationships between the serum neutralization titres of TOSV, SFSV and GRPV. No significant correlation was found between TOSV and GRPV (*p* = 0.9876) or between SFSV and GRPV (*p* = 0.5692). These results indicate that the GRPV-positive samples are not linked to the TOSV and SFSV samples, which suggests a distinct immunological response with no evidence of cross-reactivity (Supplementary Table 4).

### Prevalence by district

**Figure 3.**
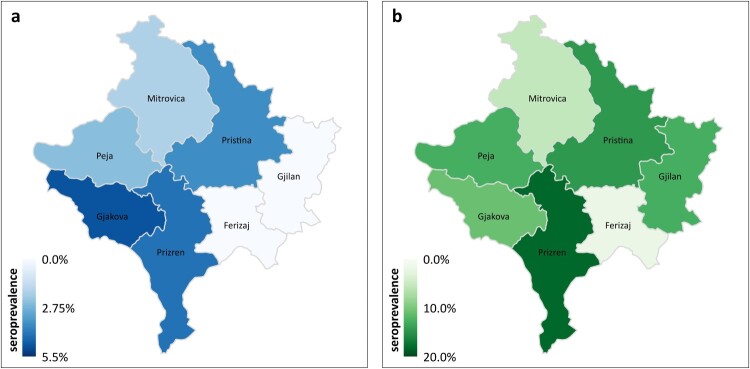
GRPV seroprevalence in dogs (a) and humans (b) by district in the Republic of Kosovo.

The seroprevalence of GPRV in dog sera ranged from 0.0% in Ferizaj and Gjilan, 2.5% (95% CI: 0.1–14.7) and 2.6% (95% CI: 0.1–15.4) in Mitrovica and Peja, respectively, 4.0% (95% CI: 0.7–14.9) in Pristina to 5.0% (95% CI: 0.9–18.2) in Prizren and 5.1% (95% CI: 0.9–18.6) in Gjakova (Figure 3(a)).

In humans, seroprevalence of GRPV varied from 2.3% (95% CI: 0.1–13.5) in Ferizaj, 6.4% (95% CI: 1.7–18.6) in Mitrovica, 12.5% (95% CI: 0.7–53.3) in Gjakova, 13.3% (95% CI: 2.3–41.6) in Peja and Gjilan, 14.5% (95% CI: 11.3–18.2) in Pristina to 17.5% (95% CI: 11.2–18.3) in Prizren (Figure 3(b)).

Due to the small number of samples in some municipalities, we refrained from conducting a detailed analysis (Supplementary Table 5). The prevalence rates by municipality are presented in Supplementary Figure 4.

### In vitro viral growth kinetics

**Figure 4.**
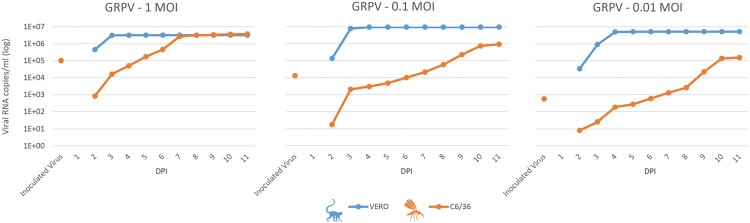
In vitro growth kinetics of Grapi virus from Kosovo in African green monkey kidney cell line (Vero) in blue and *Aedes albopictus* larvae cell line (C6/36) in orange. Cells were infected with a multiplicity of infection (MOI) of 1, 0.1 and 0.01; supernatants were collected every 24 h for 11 days post-infection (DPI). Viral genome copies were measured at indicated time points by specific real-time reverse transcription PCR.

The in vitro growth kinetics of GRPV in Vero cells showed a significant increase in viral loads from the second day post infection (DPI), reaching a plateau at 3 DPI for 1 and 0.1 MOI and at 4 DPI for 0.01 MOI. The maximum viral titre was between 10^6 and 10^7 viral copies per mL on Vero cells. On the other hand, the growth kinetics of GRPV in C6/36 mosquito cell lines showed a slower but constant increase, confirming viral replication in mosquito cells (Figure 4). For 1 MOI, the viral load increased steadily up to 7 dpi, reaching a titre of 5 × 10^6 before stabilizing. For 0.1 and 0.01 MOI, the viral titre continued to increase up to 10 days before reaching the plateau, with viral titres of 10^6 and 10^5, respectively.

## Discussion

The discovery of GRPV, a proposed novel member of the *Phlebovirus adanaense* species, in Kosovo adds critical insights into the evolving landscape of VBDs in the Balkan Peninsula. This region, characterized by its unique biogeographical position bridging Europe and Asia, has long been recognized as a hotspot for emerging pathogens due to its climate, biodiversity, and anthropogenic factors. The identification of GRPV underscores the dynamic interplay between ecological niches, vector competence, and viral evolution in shaping public health risks.

In Kosovo, the humid continental climate, marked by warm summers and dense vegetation, provides ideal breeding conditions for sand flies, particularly *Ph. perfiliewi* and *Ph. neglectus*, which dominate the local phlebofauna. These species are established vectors for phleboviruses such as TOSV and BREV, both previously detected in neighbouring Balkan countries. The absence of prior SbPV reports in sand flies from Kosovo, despite serological evidence of human and animal exposure, highlights a surveillance gap now addressed by this study. The detection of GRPV in four districts (Mitrovica, Peja, Prizren, Gjakova) is coherent with regions of high sand fly abundance, as recently documented [14]. These areas, characterized by rural landscapes and forested zones like the Grapi forest, likely facilitate sustained enzootic transmission cycles.

The role of the Balkan Peninsula as a cross-border corridor for wildlife and human migration further amplifies the risk of pathogen spillover. GRPV genetic proximity to BREV (97.55–98.70% nucleotide identity across segments) and other *Phlebovirus adanaense* members suggests regional viral diversification driven by shared vectors and reservoir hosts. The predominance of *Ph. perfiliewi* as the GRPV vector mirrors findings from North Macedonia, where BREV was isolated in the same species, indicating a possible vector-specific adaptation [6]. This ecological consistency across borders underscores the need for transnational surveillance networks to monitor phlebovirus spread.

The tripartite genome structure of GRPV conforms to the canonical phlebovirus organization, but possible partial intrasegmental recombination analyses revealed intriguing evolutionary dynamics. GRPV is closely related to BREV, ADAV and PONV [24]. All were isolated from sand flies of the subgenus *Larroussius* in North Macedonia, Turkey and Italy, respectively, which highlights the wide geographical distribution of viruses belonging to *Phlebovirus adanaense* species. While the L and S segments showed phylogenetic clustering with BREV and ADAV, the M segment exhibited divergent ancestry, potentially linked to MVV. Such segment-specific reassorting events, common in segmented RNA viruses, may affect viral fitness. This type of evolutionary event has been documented in the New World among viruses within the *Phlebovirus candiruense* species [25]. Comparable events have also been observed in the Old World, involving Granada and Arrabida viruses within the *Phlebovirus massiliaense* species, particularly in association with former Salehabad complex viruses such as PONV and MVV, as well as possible reassortant strains like BREV and ZABAV [6,26–28]. For instance, the glycoproteins (Gn/Gc) mediate host cell entry; recombination here could alter tropism or pathogenicity. Since glycoproteins are crucial for recognizing and attaching to host or vector cell surfaces, reassortment events may affect vector specificity as well as the immune response generated in human or animal hosts [29]. Although reassortment is most frequently observed in the M segment, the potential for genetic exchange in the other segments should not be overlooked [27]. Reassortment events, while predominantly involving the M segment, have also been reported in the S segment [30,31].

The high genetic identity between GRPV and BREV raises questions about their ecological coexistence. These viruses may represent sympatric variants circulating within the same vector populations, or they may instead occupy distinct ecological niches. The detection of GRPV in Kosovo, alongside BREV in North Macedonia, suggests localized viral radiation shaped by geographic barriers or host preferences. Further metagenomic studies comparing viral strains across the Balkans could elucidate these patterns.

Our morphological and molecular identification of GRPV-positive samples are in line with a recent entomological survey in Kosovo, which reported a high abundance of *Ph. perfiliewi* in GRPV positive regions of the country [14] . Since all GRPV-positive pools were identified as *Ph. perfiliewi*, this species is most likely the principal vector of GRPV, similarly to its role in transmitting BREV [6]. Nevertheless, the sequencing depth may not have been sufficient to detect potential low-abundance species that could have been overlooked, including *Ph. neglectus,* the second most abundant sand fly species. GRPV minimum infection rate (∼2%) is higher than that of BREV [6], indicating high circulation of the virus in Kosovo.

We identified a relatively high percentage of seroneutralizing antibodies against GRPV in humans and dogs (13.0% and 2.7%, respectively), confirming that GRPV can potentially cause infection in humans and dogs. Xhekaj et al. [14]identified *Ph. perfiliewi* as a multi-host feeding species in Kosovo, with blood meals including cattle, dogs, and humans. The total neutralizing antibody rates against GRPV was higher than both BREV (3.3%) in North Macedonia and ADAV (0.7%) in Turkey in humans and similar to BREV (2.4%) in North Macedonia in dogs [6,32]. GRPV infection rates were similar between women (13.3%) and men (12.5%), differing from BREV results in North Macedonia, where only males were found to be infected. This discrepancy may be due to sample size limitations [6]. The highest seropositivity in adolescents (11–20 years: 27.3%) may reflect behavioural factors (e.g. outdoor activities) which increase exposure to sand flies. Conversely, the sharp decline in older age groups (2.8% in 51–60-year-olds) could indicate waning antibodies or reduced exposure over time. Whether GRPV can cause human disease remains unknown but the high prevalence rates justifies conducting specific studies to address this question in Kosovo.

Interestingly, the seropositivity rates doubled in dogs with compromised health state (4.8%) compared to those healthy ones (2.4%), raising the question of whether GRPV could be pathogenic for dogs and whether comorbidities contribute to higher seropositivity levels in sick dogs. Additionally, seroneutralization tripled in purebred dogs (4.5%) compared to mixed-breed dogs (1.7%). The difference in infection rates between mixed-breed and purebred dogs could be due to genetic diversity, with mixed-breed dogs having stronger immune systems. Purebred dogs may be more prone to inherited health issues due to selective breeding [33]. Environmental factors, owner behaviour, and differences in veterinary care may also influence the rates. Additionally, sample size limitations could affect the results.

GRPV exhibits no serological cross-reactivity with TOSV or SFSV, indicating that GRPV-neutralizing antibodies result from specific past infections rather than cross-reactive immune responses.

GRPV rapid replication in Vero cells (10⁶–10⁷ copies/mL by 3–4 dpi) contrasts with slower proliferation in C6/36 mosquito cells, suggesting preferential adaptation to mammalian hosts This pattern is consistent with findings for BREV and ZABAV [6]. However, previous attempts to replicate other SbPVs in mosquito cells were unsuccessful for SFSV, SFNV, TOSV, and Ntepes virus [34]. The replication of GRPV in C6/36 cells, along with its genetic relationship to the mosquito-borne Arumowot and Odrenisrou viruses, raises the question of whether GRPV could also be transmitted by mosquitoes [35] and whether the evolutionary origin of the Salehabad virus serocomplex lies in mosquito-borne phleboviruses.

GRPV ability to replicate in both cell types underscores its ecological versatility. So far, the absence of clinical data linking GRPV to disease in humans or animals remains a critical gap. Experimental infections in animal models could clarify its pathogenicity, while diagnostic studies on febrile patients in Kosovo may establish clinical correlations.

This study has several limitations. Human sera were obtained from laboratory cohorts, which may not well represent the general population; similarly, dog sampling focused on *Leishmania*-endemic areas, potentially biasing seroprevalence estimates. While GRPV-positive sand flies were found in four districts, sparse sampling in northern Kosovo limits conclusions about countrywide distribution.

Future research should prioritize (i) longitudinal studies to track GRPV transmission dynamics seasonally, (ii) pathogenicity assays in animal models to assess virulence, (iii) expanded entomological surveys to map the role of vector species in virus maintenance, and (iv) implementation of clinical studies in unexplained febrile illness to assess the potential role of GRPV.

The discovery of GRPV in Kosovo illuminates the role of the Balkan Peninsula as a crucible for emerging phleboviruses. Its genetic proximity to BREV, significant human seroprevalence, zoonotic transmission potential, and rapid replication in mammalian cells position GRPV as a possible public health concern. While questions remain about its clinical impact, this study highlights the necessity of proactive, interdisciplinary surveillance to mitigate the threats posed by cryptic arboviruses.

## Supplementary Material

Supplementary Table 4.docx

Supplementary Table 5.xlsx

Supplementary Figure 1.jpg

Supplementary Figure 2.png

Supplementary Table 3.xlsx

Supplementary Table 7.xlsx

Supplementary Table 1.xlsx

Supplementary Figure 4.jpeg

Supplementary Table 6.xlsx

Supplementary Figure3.png

Supplementary Table 2.xlsx

## Data Availability

All data are included in the article and Supplementary Material.
