# Supplementary figures and images for: Sand fly–associated phlebovirus with evidence of neutralizing antibodies in humans and dogs in Kosovo
Source: Emerg Microbes Infect. 2025 Dec 22;15(1):2608407. doi: 10.1080/22221751.2025.2608407 (PMC12798671; doi:10.1080/22221751.2025.2608407)

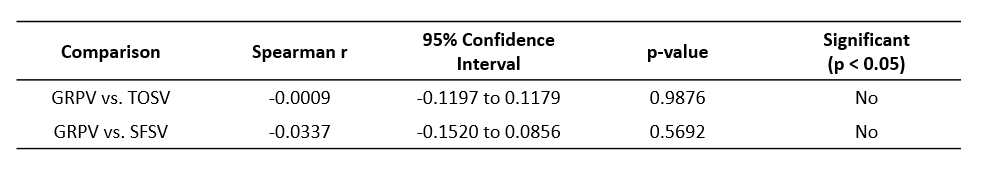

Supplement: Supplementary Table 4.docx [file TEMI_A_2608407_SM4487.docx]

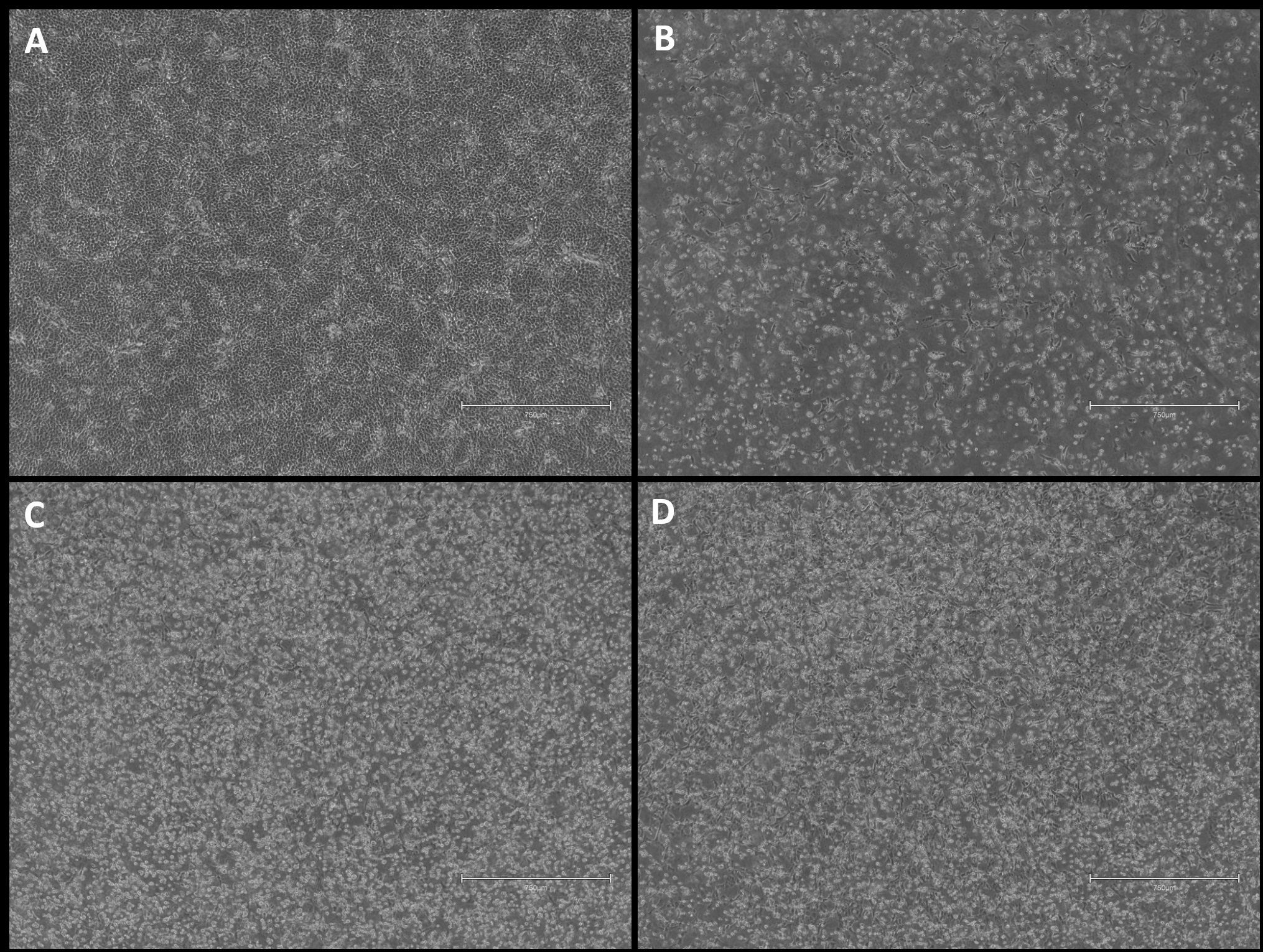

Supplement: Supplementary Figure 1.jpg [file TEMI_A_2608407_SM4485.jpg]

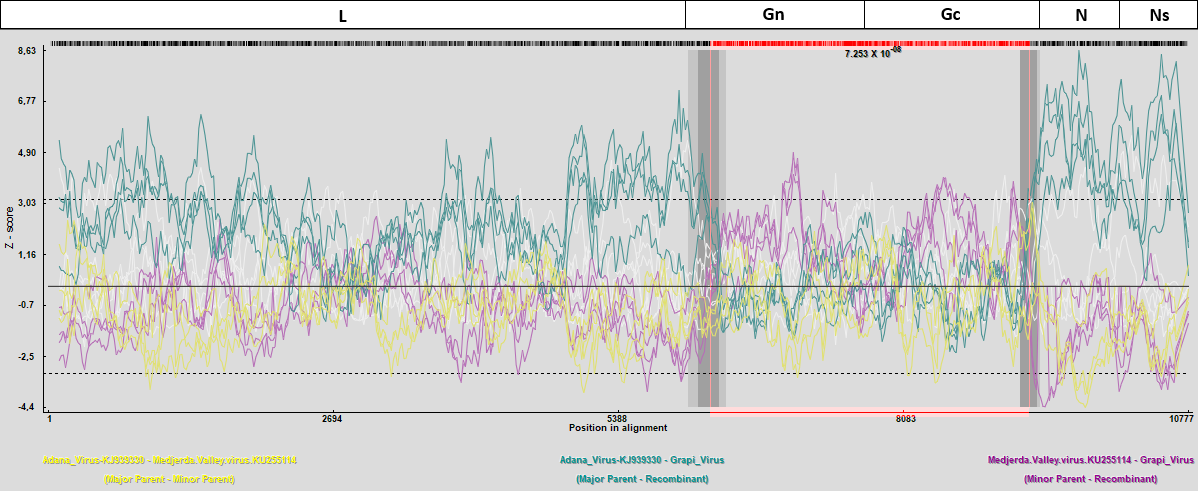

Supplement: Supplementary Figure 2.png [file TEMI_A_2608407_SM4484.png]

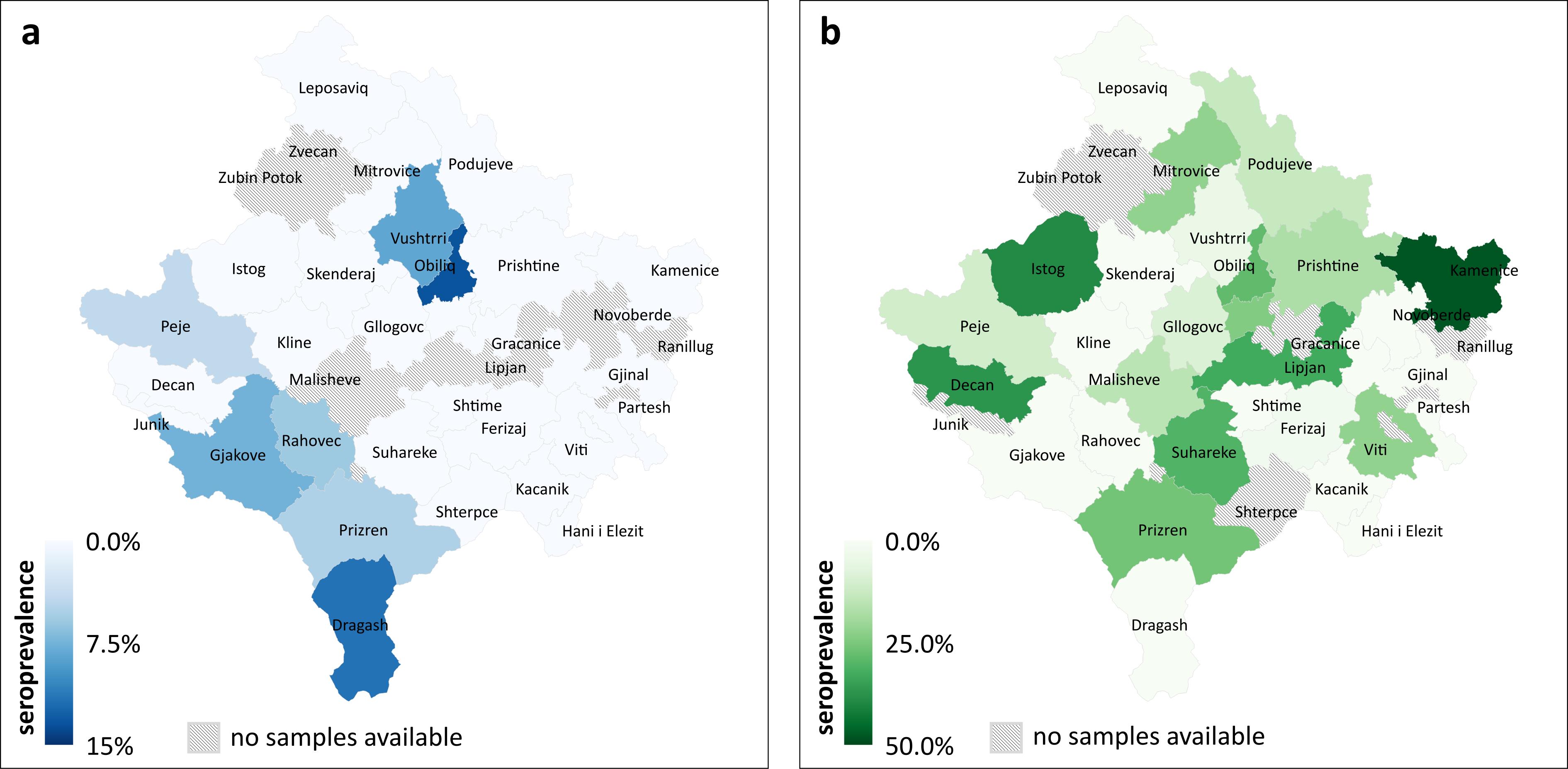

Supplement: Supplementary Figure 4.jpeg [file TEMI_A_2608407_SM4480.jpeg]

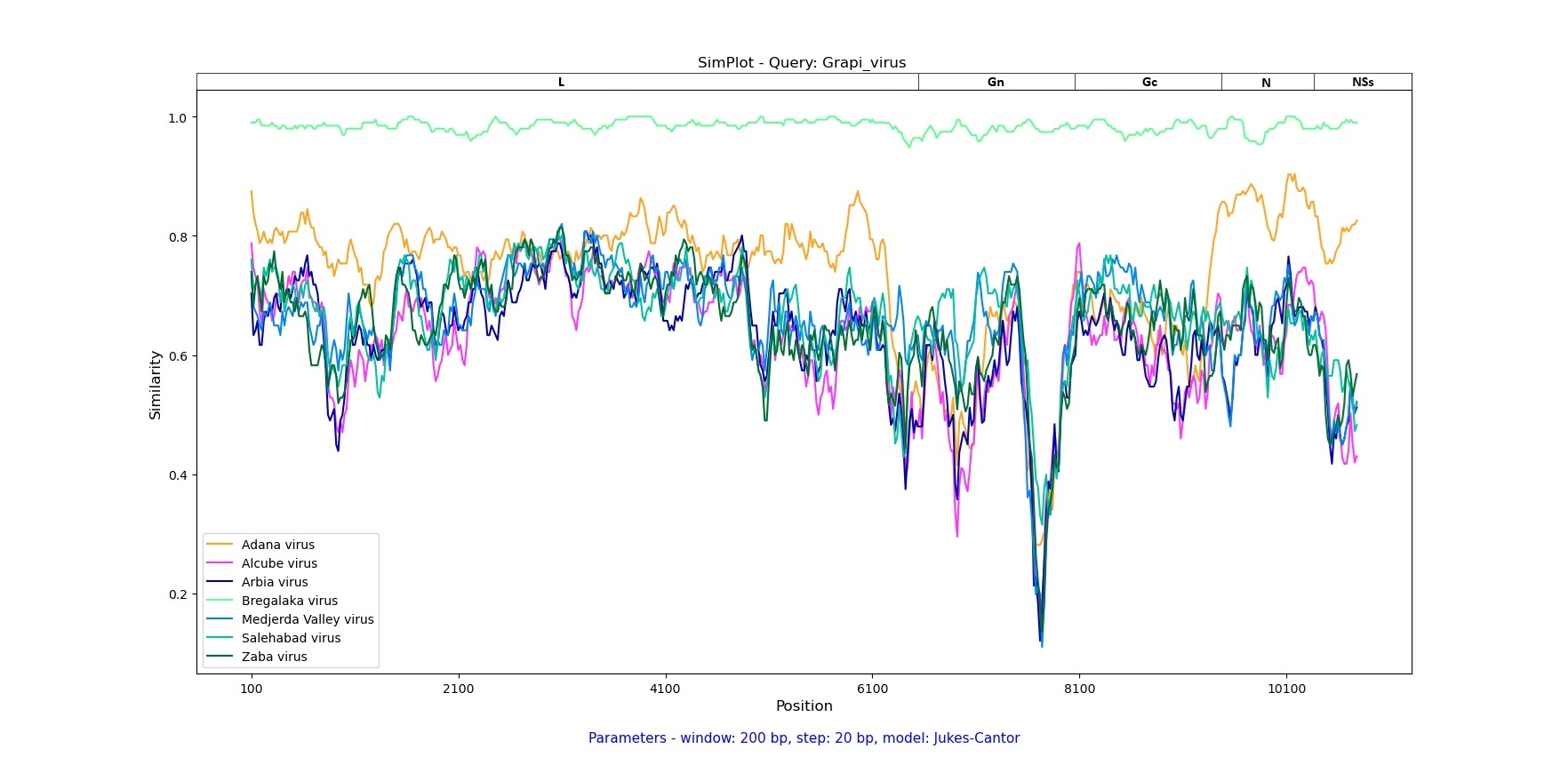

Supplement: Supplementary Figure3.png [file TEMI_A_2608407_SM4478.png]
